# Disease Concept-Embedding Based on the Self-Supervised Method for Medical Information Extraction from Electronic Health Records and Disease Retrieval: Algorithm Development and Validation Study

**DOI:** 10.2196/25113

**Published:** 2021-01-27

**Authors:** Yen-Pin Chen, Yuan-Hsun Lo, Feipei Lai, Chien-Hua Huang

**Affiliations:** 1 Graduate Institute of Biomedical Electronics and Bioinformatics National Taiwan University Taipei City Taiwan; 2 Department of Emergency Medicine National Taiwan University BioMedical Park Hospital Hsinchu County Taiwan; 3 Department of Emergency Medicine National Taiwan University Hospital Taipei City Taiwan; 4 Department of Applied Mathematics National Pingtung University Pingtung City Taiwan

**Keywords:** electronic health record, EHR, disease embedding, disease retrieval, emergency department, concept, extraction, deep learning, machine learning, natural language processing, NLP

## Abstract

**Background:**

The electronic health record (EHR) contains a wealth of medical information. An organized EHR can greatly help doctors treat patients. In some cases, only limited patient information is collected to help doctors make treatment decisions. Because EHRs can serve as a reference for this limited information, doctors’ treatment capabilities can be enhanced. Natural language processing and deep learning methods can help organize and translate EHR information into medical knowledge and experience.

**Objective:**

In this study, we aimed to create a model to extract concept embeddings from EHRs for disease pattern retrieval and further classification tasks.

**Methods:**

We collected 1,040,989 emergency department visits from the National Taiwan University Hospital Integrated Medical Database and 305,897 samples from the National Hospital and Ambulatory Medical Care Survey Emergency Department data. After data cleansing and preprocessing, the data sets were divided into training, validation, and test sets. We proposed a Transformer-based model to embed EHRs and used Bidirectional Encoder Representations from Transformers (BERT) to extract features from free text and concatenate features with structural data as input to our proposed model. Then, Deep InfoMax (DIM) and Simple Contrastive Learning of Visual Representations (SimCLR) were used for the unsupervised embedding of the disease concept. The pretrained disease concept-embedding model, named EDisease, was further finetuned to adapt to the critical care outcome prediction task. We evaluated the performance of embedding using t-distributed stochastic neighbor embedding (t-SNE) to perform dimension reduction for visualization. The performance of the finetuned predictive model was evaluated against published models using the area under the receiver operating characteristic (AUROC).

**Results:**

The performance of our model on the outcome prediction had the highest AUROC of 0.876. In the ablation study, the use of a smaller data set or fewer unsupervised methods for pretraining deteriorated the prediction performance. The AUROCs were 0.857, 0.870, and 0.868 for the model without pretraining, the model pretrained by only SimCLR, and the model pretrained by only DIM, respectively. On the smaller finetuning set, the AUROC was 0.815 for the proposed model.

**Conclusions:**

Through contrastive learning methods, disease concepts can be embedded meaningfully. Moreover, these methods can be used for disease retrieval tasks to enhance clinical practice capabilities. The disease concept model is also suitable as a pretrained model for subsequent prediction tasks.

## Introduction

### Background

Diagnosing a disease is like putting together a puzzle. When more pieces match, we can decipher the picture more easily. Or, if we have a reference picture of the puzzle, matching the pieces can be accomplished more easily. Many “puzzles” have been collected in electronic health records (EHRs), which contain abundant information about patients and diseases and represent a treasure trove for medical research. However, EHRs are challenging to use effectively due to the heterogeneity of the data types they can contain [1-3].

Sometimes, doctors in emergency departments must develop treatment plans based on limited information from seriously ill patients. In such cases, only demographic information, vital signs (eg, blood pressure, respiratory rate, and blood oxygen saturation), and major discomfort information can be obtained. However, a patient’s medical history may be collected through old records in the hospital database, and these records can provide doctors with vital information that can help them to identify serious diseases [4]. Based on the doctor’s knowledge and experience, the patient’s information can characterize the disease, enable the doctor to diagnose the patient, and allow the doctor to develop an appropriate treatment plan to prevent the disease from worsening.

Doctors can make great use of the rich information contained within patients’ EHRs. However, it is almost impossible for EHRs to be of help to doctors if they are not organized. Full use of the medical information contained within EHRs in clinical practice greatly enhances doctors’ abilities to treat patients [3,5,6].

The International Statistical Classification of Diseases and Related Health Problems 10th Revision (ICD-10) code [7] is a well-established classification system for diseases that contains approximately 70,000 diagnoses. These diagnoses are highly specific and contain meaningful words that can be used for patient medical records. ICD codes can easily be used as keywords to search for an EHR. However, it is difficult to search for records without the ICD code of a patient.

With the rapid development of natural language processing and deep learning during this decade, several models have been released to manage EHRs [1,8-10] using several primary tasks, including disease classification, prediction of clinical events, and concept embedding [2]. Due to the lack of gold standard label data for model training, concept embedding is critical to all EHR-related tasks [1,2,11,12]. Concept embedding can be regarded as a pretrained model that can be finetuned with limited label data for further event prediction, disease classification, and detection, including the diagnosis of diseases. Concept embedding can also be used for data retrieval [13,14], and can be further integrated into medical software, web services, and apps used by patients for disease screening purposes.

Therefore, the concept-embedding model is essential to EHR research because it can organize rich medical experience and knowledge. We aim to develop a disease embedding model that can cluster disease patterns based on initial data (eg, demographic information, vital signs, chief complaint, and medical history) collected from patients who have just arrived at a hospital.

### Related Works

Structural data (eg, demographic information, blood pressure, and heart rate) and free text records are collected in an EHR. In traditional medical research, free text records are more difficult to analyze, and significant human resources are required to classify free text into tables using rule-based methods [1,2,10]. With the vigorous development of deep learning, several language models for extracting features from free text records have become available [15-17]. Long short-term memory [18] is a model architecture that can feed sequence data and properly ignore input or memory history data to extract accurate features. It was widely used on natural language processing tasks before Transformer [19] was proposed.

Transformer contains the multihead self-attention model, which can learn the attention (attention can be regarded as relevance) of words within the text. Transformer was released to perform translation tasks and speed up learning by feeding all data simultaneously (instead of feeding them one-by-one as is the case with long short-term memory). Bidirectional Encoder Representations from Transformers (BERT) [16] is an extension of Transformer, which has achieved excellent performance ratings in several competitions. The first version of BERT contained only Transformer’s encoder, which pretrained the embedding of subwords (words broken into pieces) unsupervised through a cloze procedure and subsequent sentence prediction. Because translation was the original goal of Transformer, the multilingual pretrained BERT is suitable for EHR research in Taiwan because Taiwanese medical records contain English and Chinese.

Previous methods for concept embedding of the EHR model include long short-term memory, convolutional neural networks, and autoencoders [1,2,11,12]. An autoencoder is a widely used unsupervised method for concept embedding. It contains an encoder that converts the original input data into a hidden embedding vector, while the decoder reconstructs a result similar to the input data from the hidden embedding vector. In the EHR field, autoencoders outperform traditional machine learning methods [1]. However, the risk of reidentification is a critical issue for autoencoders. Some studies exist on protecting patient privacy with autoencoders [20,21]. However, if the autoencoder is not designed to protect privacy, there is still a risk of reidentification because the decoder is designed to minimize the difference between the original data and the output.

In the EHR field, the contrastive self-supervised learning methods that belong to unsupervised learning, including Deep InfoMax (DIM) [22] and Simple Contrastive Learning of Visual Representations (SimCLR) [23], may be superior. DIM adopted discriminators (replacing the autoencoder’s decoder) that maximize mutual information between the input data and the embedding vectors. SimCLR minimizes the embedding distance within groups while maximizing the embedding distance among groups. Compared with autoencoders, contrastive self-supervised learning methods demonstrate superior results when performing classification tasks [22], even at the level of supervised learning [23], but no decoder model produces the risk of reidentification.

Due to the vectorization of objects of interest, disease concept-embedding can be used for retrieval [24]. Vector representation of big data can be operated simultaneously using linear algebra methods [25]. EHR-based clinical information retrieval [3,13,26,27] has been proposed. One study [13] focused on creating test sets for information retrieval research. Two studies [3,26] used keyword-based information retrieval systems, which did not use the semantic analysis method and could not identify negation semantics. Another study [27]developed a document-level semantic-based query recommendation search engine. Furthermore, these studies only focused on EHR-related retrieval methods that cannot be directly applied to predictive models. No objective score or standard exists to evaluate the performance of the concept-embedding model or the information retrieval [3]. However, visualization is a widely used method for evaluating the performance of concept embedding [10]. The t-distributed stochastic neighbor embedding (t-SNE) [28] method reduces the embedding’s dimensionality to visualize the clustering effects of unsupervised learning. Although not objective, another evaluation method is sampling each cluster group and determining its topic [13,29]. Therefore, we can use a patient’s data to retrieve neighbor samples and verify the consistency of the samples’ concepts. Furthermore, if a new patient data is provided, concept embedding can be used to retrieve the top five most similar data, after which we can evaluate the retrieval capabilities.

In some papers, subsequent applications of classification have been adopted [2]. For emergency department employees, a patient’s condition severity and emergency information are most relevant. We introduced a label for critical care outcome, defined as either (1) an intensive care unit admission or (2) death in the hospital after an emergency department visit [30-35]. This outcome was used to finetune the pretrained embedding model for the classification task. Furthermore, we compared the performance of the embedding model with that of state-of-the-art models.

### Objective

This study aims to create a model that uses limited patient information to extract disease concept-embedding for disease retrieval and classification tasks. Accordingly, we present a disease concept-embedding method, the EDisease model, based on Transformer and the contrastive self-supervised learning method.

## Methods

### Materials

A sample of 1,040,989 emergency department visits was collected from the National Taiwan University Hospital Integrated Medical Database, a private EHR data set. The sample included the patients of National Taiwan University Hospital (one medical center in Taipei City and two regional hospitals in Hsinchu City and Yunlin County), who visited the hospital from January 1, 2013 to December 31, 2017. This study was approved by Research Ethics Committee B at National Taiwan University Hospital (201902078RINB).

In the data preprocessing stage, if the chief complaint was missing data or the structural data were unreasonable (eg, blood pressure was higher than 300 mmHg, diastolic blood pressure was higher than systolic blood pressure, heart rate was higher than 250 beats/minute, respiration rate was higher than 100 times/minute, body temperature was higher than 48°C or lower than 20°C, and body weight and height were higher than 400kgs and 250cms, respectively), the samples were discarded. In this stage, structural data with missing values were retained.

Individual patients may have visited the hospital several times during the specified period, and each visit corresponded to one account. Thus, each patient’s ID number may have been associated with multiple EHRs. In the National Taiwan University Hospital, if a patient visits more than once within 24 hours, the last account number can be retained for reuse using another revisit flag. In this study, we retained only the last sample with the same account but different revisit flags. Furthermore, for each ID number, there are several medical records in the EHR, and we used the triage time to filter future records.

**Figure 1:**
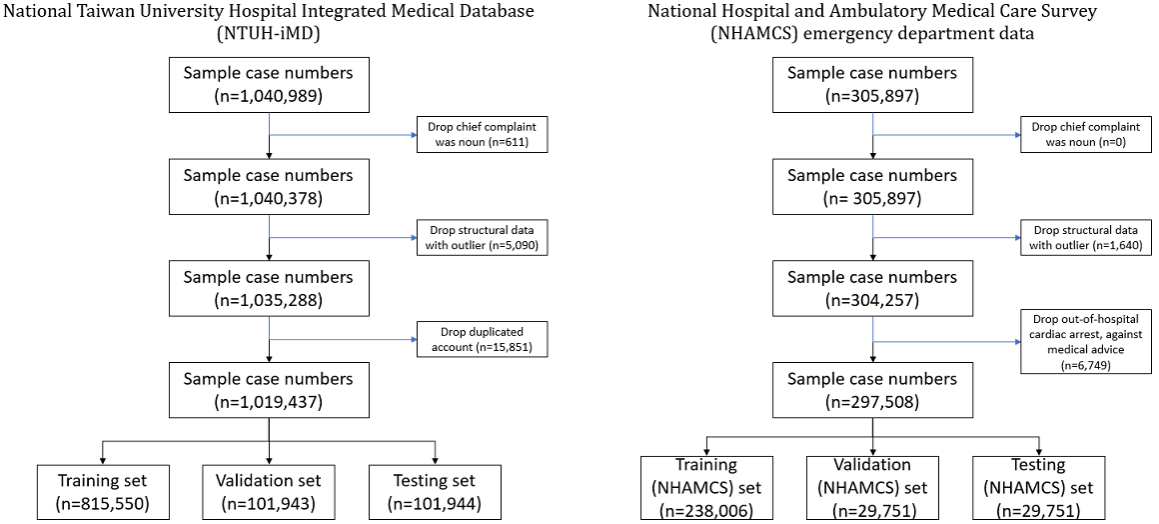
Data analysis flowcharts.

We then split the sample of 1,019,437 visits into sizes of 815,550 (80.0%), 101,943 (10.0%), and 101,944 (10.0%) for the training, validation, and test sets, respectively (Figure 1).

We also collected National Hospital and Ambulatory Medical Care Survey emergency department data from 2007 to 2017 [36]. This survey data is available in a public EHR database provided annually by the Centers for Disease Control and Prevention National Center for Health Statistics. We preprocessed the structured data using the same preprocessing method described previously and excluded patients with death on emergency department arrival, or those who left before being seen by a practitioner or against medical advice. Structured chief complaints and comorbidities were converted into free text as the chief complaints and past medical history, respectively. For the National Hospital and Ambulatory Medical Care Survey data, we split the 297,508 visit sample into sizes of 238,006 (80.0%), 29,751 (10.0%), and 29,751 (10.0%) for training, validation, and test sets, respectively (Figure 1).

The means and standard deviations were counted after discarding the missing data. Moreover, critical care outcome was selected as the outcome. Critical care outcome refers to the union of intensive care unit admission and death. We distinguished the definitions of intensive care unit admission and death for each data set. In the National Taiwan University Hospital Integrated Medical Database, intensive care unit admission is defined as whether an intensive care unit record existed within three days after a patient’s emergency department visit. Death is defined as whether a death record existed within three days after a patient’s emergency department visit [30,31]. In the National Hospital and Ambulatory Medical Care Survey, intensive care unit admission is defined as direct admission to an intensive care unit, and death is defined as in-hospital death (Table 1).

Emergency staff work in shifts to treat emergency patients promptly. Therefore, an alert system for impending emergency events is useful. Consequently, we chose a three-day interval as the cut-off point for disease progression, resulting in the outcome in the National Taiwan University Hospital Integrated Medical Database being defined as any critical event within three days after an emergency department visit. In contrast, our definition of critical care outcome in the National Hospital and Ambulatory Medical Care Survey was identical to that of the reference [32-34].

**Table 1:** Structural information and outcomes.

Variable		National Taiwan University Hospital Integrated Medical Database				NHAMCS^a^
		Medical center (Taipei City) (n=541,366)	Regional hospitals (Hsinchu City) (n=233,921)	Regional hospitals (Yunlin County) (n=244,150)	Total (N=1,019,437)	Emergency department data (n=297,508)
					
Age, mean years (SD)		43.1 (27.0)	42.2 (27.2)	46.9 (26.0)	43.7 (26.9)	37.4 (24.0)
Gender (male), n (%)		266,891 (49.3)	118,815 (50.8)	129,221 (52.9)	514,927 (50.5)	135,147 (45.4)
**Vital signs**						
	Systolic blood pressure, mmHg (SD)	133.0 (26.9)	140.2 (28.5)	136.2 (26.2)	135.4 (27.3)	132.9 (23.5)
	Diastolic blood pressure, mmHg (SD)	77.8 (15.2)	77.7 (15.4)	80.3 (17.2)	78.3 (15.8)	77.7 (14.6)
	Heart Rate, BPM^b^ (SD)	97.6 (26.8)	97.0 (26.0)	90.0 (20.8)	95.8 (25.6)	91.1 (22.8)
	Saturation, % SpO_2_ (SD)	97.0 (2.8)	97.1 (3.0)	97.6 (5.6)	97.2 (3.64)	97.3 (6.2)
	Respiratory rate, BrPM^c^ (SD)	19.9 (3.7)	20.4 (2.5)	17.6 (2.4)	19.5 (3.4)	19.3 (4.4)
	Body temperature, °C (SD)	37.1 (0.9)	37.1 (1.0)	36.9 (1.0)	37.1 (1.0)	36.8 (0.6)
	Body height, cm (SD)	151.6 (27.9)	153.4 (25.5)	157.4 (20.4)	152.8 (26.6)	N/A^d^
	Body weight, kg (SD)	53.3 (22.3)	47.0 (25.1)	54.4 (21.9)	52.5 (22.8)	N/A
	Pain index, value (SD)	2.6 (3.2)	3.1 (3.3)	2.3 (2.9)	2.7 (3.2)	4.8 (3.7)
	Glasgow coma scale, value (SD)	14.8 (1.3)	14.7 (1.4)	14.6 (1.7)	14.7 (1.4)	14.5 (2.1)
	Eye response, value (SD)	4.0 (0.3)	4.0 (0.3)	3.9 (0.4)	4.0 (0.3)	N/A
	Verbal response, value (SD)	4.9 (0.5)	4.9 (0.6)	4.8 (0.7)	4.9 (0.6)	N/A
	Motor response, value (SD)	5.9 (0.4)	5.9 (0.5)	5.9 (0.7)	5.9 (0.5)	N/A
**Outcomes**						
	Admission to ICU, n (%)	1,958 (0.4)	336 (0.1)	613 (0.3)	2,907 (0.2)	4,375 (1.5)
	Death, n (%)	1,993 (0.4)	706 (0.3)	891 (0.4)	3,590 (0.4)	381 (0.1)
	Critical care outcome, n (%)	3,906 (0.7)	1,039 (0.4)	1,492 (0.6)	6,437 (0.6)	4,755 (1.6)

^a^NHAMCS: National Hospital and Ambulatory Medical Care Survey.

^b^BPM: beats per minute.

^c^BrPM: breaths per minute.

^d^N/A: not available.

### Model Architecture

**Figure 2:**
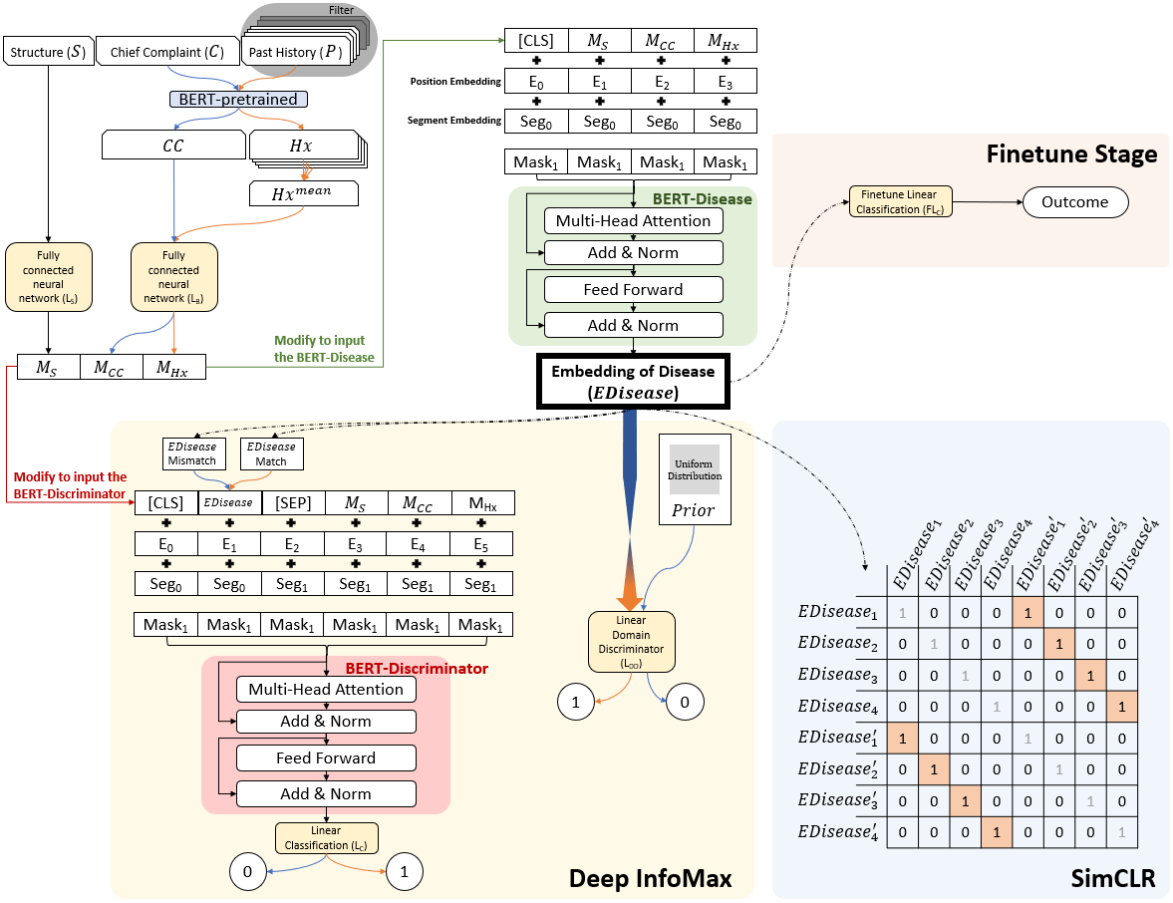
Deep-learning model architecture.

Transformer was adopted as the central architecture in this study, based on BERT with the PyTorch adaptation released by the HuggingFace team [37]. Initially, we collected structural data *S* ∈ **R^15^**, including age, gender, blood pressure, heart rate, blood oxygen saturation, respiratory rate, body temperature, height, weight, subjective pain score, and Glasgow coma scale. We filled each unavailable data point with the average value of each *S* and performed noise processing on each element in *S* with a random scale based on the standard deviation of each element [38]. The fully-connected neural network for structural data L*_S_* is a multilayer perceptron, which converts *S* to *M_S_* ∈ **R**^96^ (Figure 2). For the free text medical record data, the pretrained “bert-base-multilingual-cased” model (ie, *BERT-*pretrained) is used to extract features *CC*, *Hx*, ∈ **R**^768^ from the free text (chief complaint, *C,* and past medical history, *P*). Because the number of past medical history data points changes, we averaged the features of all *Hx* and obtain the *Hx^mean^* that represents the patient’s medical history. If there is no medical history in the EHR, we use a padding vector to fill the *Hx^mean^.* The fully-connected neural network *L_B_* for *BERT* is a multilayer perceptron that converts *CC* and *Hx^mean^* to *M_cc_* and *M_Hx_* ∈ **R**^96^, respectively.

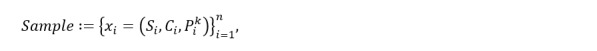



*n* is the total number of samples, *k* denotes the numbers of medical history.

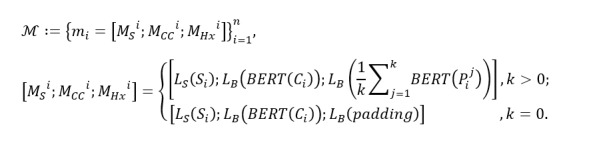



We embed the diseases to concepts by adopting another BERT architecture as *BERT-*Disease (*β_ED_*), a 12-layer Transformer encoder with 12 self-attention heads and a hidden size of 96. We concatenate *M_S_, M_cc_*, *M_Hx_* and modify it with a leading head [CLS] as the input embedding. Position embedding indicates each element’s position in the input embedding sequence, and segment embedding indicates the level to which the element belongs [16]. All segment embeddings are of the same level at this stage, but we reserved this settable variable for the subsequent work in this study. We obtain the sum of input embedding, position embedding, and segment embedding as the input of *β_ED_*.

The *Mask* ∈ {0,1} indicates whether the element is valid (1) or invalid (0). For example, some patients did not have any medical history records in their EHRs. In these cases, the padding *Hx^mean^* will be used as the input; however, the padding element should be ignored. Furthermore, when we gathered more information from some patients, *Mask* enabled us to ignore other padding elements and is, therefore, essential to this extensible model. We also used the random mask on *M_Hx_* for the augmentation of *M* to improve the ability to embed the disease concept in a state with less information. This augmentation of a random history mask is also adopted in the subsequent work with another modification *M*. In this part, we obtained the embedding of disease:

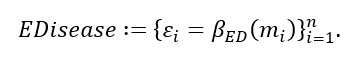



DIM was adopted to further construct accurate disease embedding. DIM’s core principle is to maximize the mutual information between the samples and embedding [22]. The mutual information on *M* and *EDisease* is as follows, where *p(m)* denotes the probability of each visit in the sample.

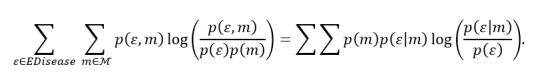



We assumed that each visit was unique. Therefore, *p(m)*,∀*m* ∈ *M* are the same constant. Moreover, we assumed the number of disease embedding data points was limited and smaller than the number of visits, such that 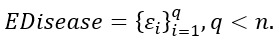. The result is that *B_ED_ : M→EDisease* is a surjective function also known as onto, so we then obtain *p*(ε) = p(ε|*M*)*p*(*M*).

We then set the function *DIM Loss* as follows and minimized it to maximize the mutual information.

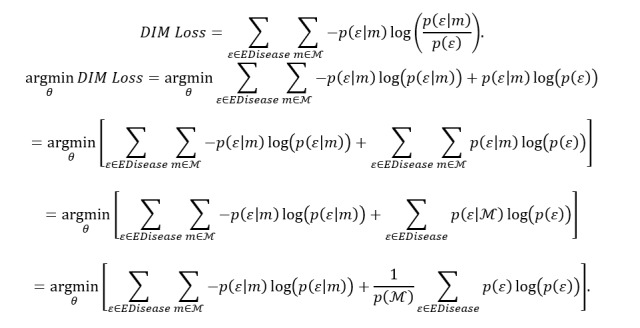



We achieved this goal by adopting another BERT architecture, *BERT-Discriminator (β_D_),* which shares the same model architecture as *B_ED_* and estimate *β_D_* (ε, *m*) = *p*(ε|*m*). In this stage, *M* is modified not only by [CLS] but also by *EDisease* and [SEP]. The [SEP] is used to separate *EDisease* and *M* and this modification is similar to “the next sentence prediction” task in BERT [16]. We used segment embedding with a level of 0 to modify the [CLS] and *EDisease* with a level of 1 to modify [SEP] and *M*. We set Y_(ε,m)_=1, when *i=j*, ∀*m*_i_,ε_j_ and Y_(ε,m)_=0, when *i*≠*j*, ∀*m_i_, ε_j_* and simplified as follows.

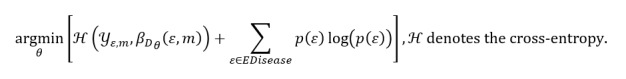



A part of *Loss* could now be discovered using the cross-entropy loss, similar to noise-contrastive estimation [39,40]. However, one part remained to be solved. We had to establish an adequate prior distribution to constrain the embedding distribution as proposed by DIM [22] and to minimize the ∑*p*(ε)log(*p*(ε)).

The function *F*(*x*) = *x* log *x* is convex [41]. Then, 

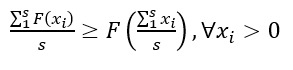



and two parts are equal if and only if every number *x_i_* is the same (it can be proved by the Lagrange multiplier), which is equivalent to the uniform distribution that can minimize *Loss*. Consequently, we adopted a uniform distribution *Prior* on [–1,1]^96^ to constrain *p*(ε).

Generative adversarial networks [42] can be used to constrain an embedded distribution to a prior distribution [22]. The final distribution 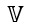 of EDisease is difficult to distinguish from the prior distribution 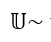 uniform distribution using a linear domain discriminator *L_DD_*. This discriminator detects whether each input distribution belongs to the prior or EDisease domain and estimates the distribution divergence of each domain. Moreover, this goal can be achieved using the generative adversarial network iteration algorithm.

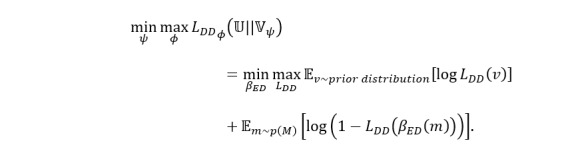



Therefore, we simplify our goal to the following equation:

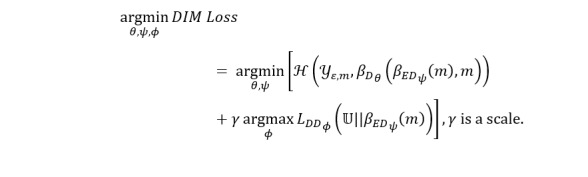



For a more robust concept embedding, we also introduced the SimCLR loss. Just as with the augmentation in SimCLR, each *m_i_* is augmented to another *m*’_*i*_ by random noise. Ideally, the cosine similarity of *B_ED_*(*m_i_*) to *B_ED_*(*m*’*_i_*) is 1 (positive sample), and the cosine similarities of *B_ED_*(*m_i_*) to *B_ED_*(*m_j_*) and *B_ED_*(*m*’_j_) are -1 (negative sample), ∀*j* ≠ *i*.

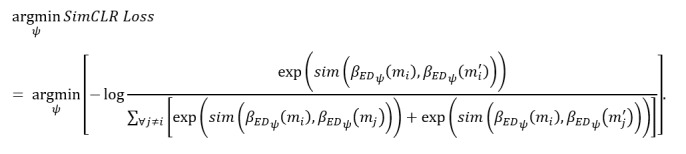



Therefore, the final loss function is:

*Loss = DIM Loss + SimCLR Loss*

In the further finetuning stage for predicting the outcome, EDisease is fed to a multilayer linear model *FL_C_* (Figure 2).

We can add an extended model for the extended information to enhance the architecture (see Multimedia Appendix 1). We demonstrated the extensible model by obtaining *M_PI_* after feeding an illness to the model and concatenating it to the vanilla *M*. The subsequent training methods are identical to those in Figure 2.

For improved performance with free text feature extraction, we again pretrained *BERT-pretrained* using the free text medical records in the National Taiwan University Hospital Integrated Medical Database. In the medical record, the chief complaint in triage was the patient’s words for their discomfort, and present illness, *I*, was the doctor’s word for the patient’s problem; thus, the similarity between the chief complaint and patient illness should, ideally, be high. Furthermore, some patients had several medical history records, and their subsets may also have been highly similar. Consequently, *CC, PI, Hx_1_^mean^, Hx_2_^mean^ ∈* ***R****^768^* were extracted by *BERT-pretrained,* the pair *CC, PI* representing the same patient’s complaints, and the pair *Hx_1_^mean^, Hx_2_^mean^* representing the same patient’s medical history. We then used the contrastive learning method used previously to pretrain *BERT-pretrained*. Due to graphic processing unit memory limitations, after again pretraining the language model, we fixed the model’s weights such that the grading of subsequent training would not change the weight of the model.

We used Adam optimization [43] for the hyperparameters and set the learning rate as 1×10^–4^, with a minibatch size of 1024. The hyperparameter γ=0.1 was chosen in this study. The source code is available on GitHub [44].

### Evaluation

We selected the training set and fed it into the model to obtain the set of disease embedding. We then used the t-SNE to reduce the dimensionality of the embedding to two for visualization. We enhanced visualization based on demographic information, hospital level, triage level, whether medical history existed, and outcome. Furthermore, we illustrated the model’s ability to cluster diseases by selecting patients from the validation set as queries to retrieve similar patients in the training set. The relevance of each result to the query was judged by a seasoned doctor, who used the number of hits on the top 5 results to score from 0-5.

Furthermore, we finetuned the model based on the outcome and compared it with the published model based on the receiver operating characteristic curve. In order to check whether the embedding could be used as a pretrained model, even if only a small amount of label data was collected, we also took 10,000 samples from the validation (National Hospital and Ambulatory Medical Care Survey) set as a small data set for finetuning. Then, we performed ablation studies by deleting each unsupervised learning method and comparing them.

## Results

We used the t-SNE method to reduce the 96-dimensional embedding to 2-dimensional embedding and enhance the visualization by gender, age (10-year intervals), hospital level, triage level, existence of a medical history, and outcome (see Multimedia Appendix 2).

**Figure 3:**
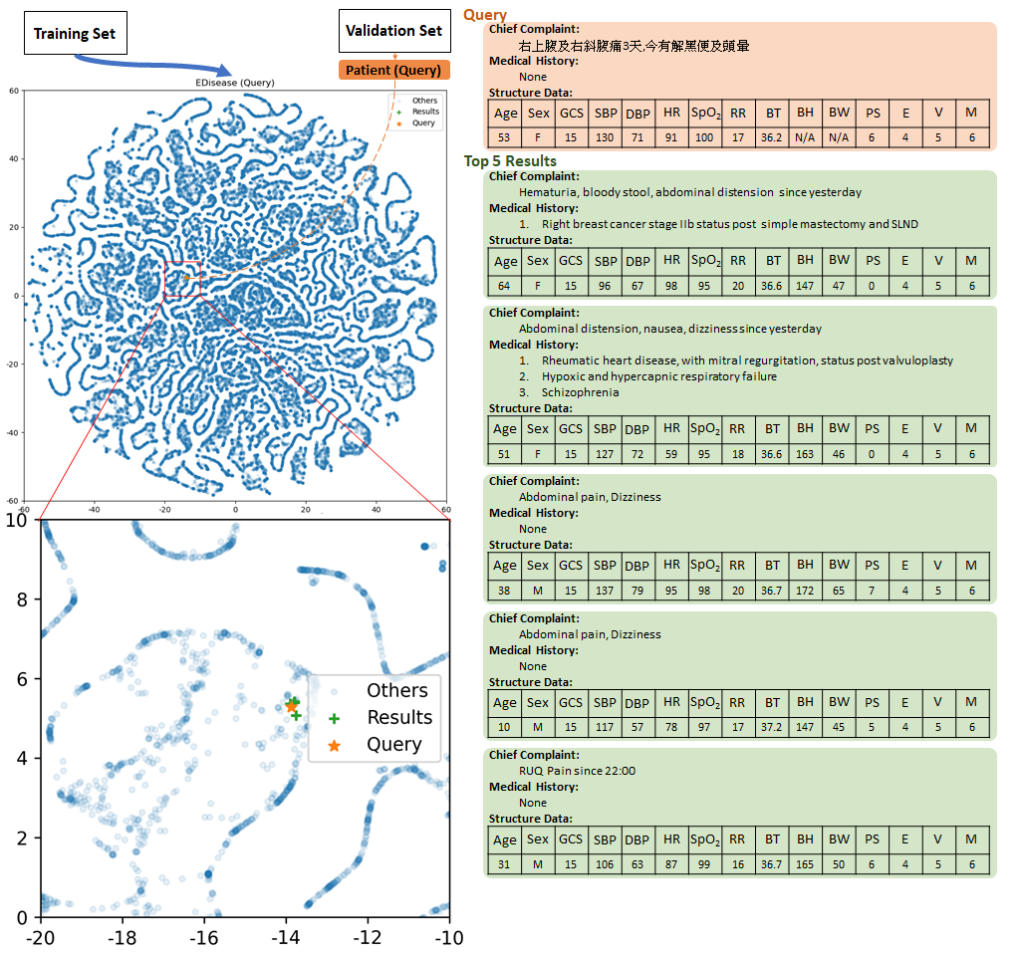
Disease Retrieval Demonstration. The query subject (orange) was a 53-year-old female patient who suffered from abdominal pain in the upper-right quadrant to right flanks for three days and noticed dizziness and tarry stool on the day of the interview. Through the retrieval, we obtained the top five similar patients (green) whose symptoms were hematuria, bloody stools, abdominal distention, abdominal pain, dizziness, and abdominal pain in the upper-right quadrant. GCS: Glasgow coma scale. SBP: Systolic blood pressure. DBP: Diastolic blood pressure. HR: Heart rate. SpO_2_: Blood oxygen saturation. RR: Respiratory rate. BT: Body temperature in Celsius. BH: Body height. BW: Body weight. PS: Pain scale. E: Eye response in Glasgow coma scale. V: Verbal response in Glasgow coma scale. M: Motor response in Glasgow coma scale. N/A : Not available.

We sampled the patients in the validation set as queries to retrieve the 5 most similar patients in the training set in the embedding space. Moreover, a doctor evaluated the relevance of 25 random query results (see Multimedia Appendix 3, Figure 3, and Multimedia Appendix 4).

The query subject (orange) was a 53-year-old female patient who suffered from abdominal pain in the upper-right quarter to right flanks for 3 days and noticed dizziness and tarry stool on the day of the interview. Through the retrieval, we obtained the 5 most similar patients (green) whose symptoms were hematuria, bloody stools, abdominal distention, abdominal pain, dizziness, and abdominal pain in the upper-right quarter.

**Figure 4:**
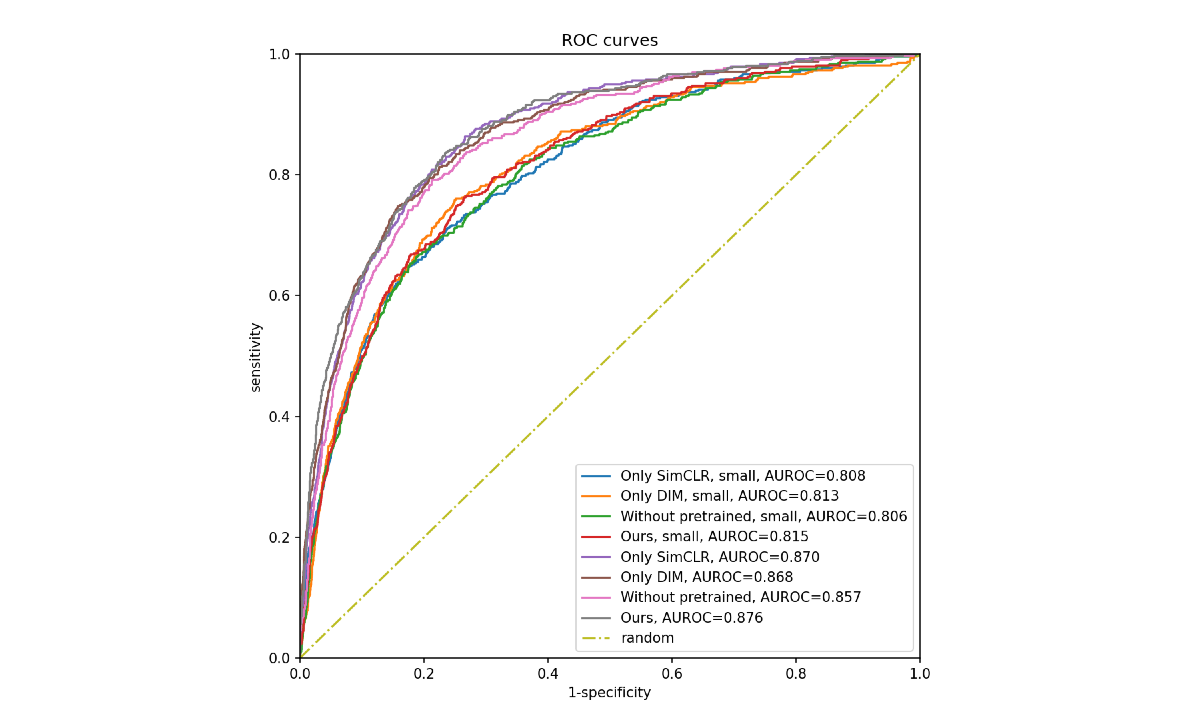
Ablation study ROC curves. Both DIM and SimCLR can improve prediction performance through pretrained embedding. Small: denotes finetuned on the smaller data set. ROC: receiver operating characteristic. DIM: Deep InfoMax. SimCLR: Simple Contrastive Learning of Visual Representations. AUROC: area under the receiver operating characteristic.

In the subsequent finetuning based on the outcome, our proposed EDisease model demonstrated the highest performance among all compared models based on the area under the receiver operating characteristic of 0.876 (Table 2, Figure 4).

**Table 2:** Area under the receiver operating characteristic results.

Model	Critical care outcome	
	NTUH-iMD^a^	NHAMCS^b^
		
Deep neural network [33] (age≤18)	N/A^c^	0.85
Deep neural network [32] (age≥18)	N/A	0.86
Hierarchical model [34]	N/A	0.84
Proposed model (without pretraining)	0.83	0.86
Proposed model	0.84	0.88

^a^NTUH-iMD: National Taiwan University Hospital Integrated Medical Database.

^b^NHAMCS: National Hospital and Ambulatory Medical Care Survey.

^c^N/A: not applicable.

The ablation study results demonstrate that our model outperformed those using fewer or no unsupervised methods, both on the large and small finetuning set. Unsurprisingly, the models finetuned on the smaller data set performed worse than those finetuned on the large National Hospital and Ambulatory Medical Care Survey training data set (Figure 4).

## Discussion

The EDisease model performed well in the disease concept-embedding task and illustrated suitable clustering performance for the disease pattern. It also demonstrated promising results in the subsequent finetuning of the outcome.

The embedding of disease is meaningful. The enhanced label with the triage level indicates that the fifth level was clustered and illustrates some influence on different classification levels (see Multimedia Appendix 2). This embedding system can be used as a disease retrieval model, which encodes queries and finds the most relevant patients and diseases. In the retrieval demonstration, the query subject was a 53-year-old female patient who suffered from abdominal pain in the upper right quarter to right flanks for 3 days and noticed dizziness and tarry stool on the day of the interview. Through the retrieval, we obtained the five most similar patients with similar symptoms that were possibly related to different diseases (Figure 3). In another example of retrieval, the query subject was a 63-year-old male patient who suffered from sudden nausea, vomiting, and weakness and felt facial numbness at work. Patients with cardiogenic symptoms or neurologic symptoms were the retrieval results (see Multimedia Appendix 4). These results are meaningful for clinical practices, such as treating cardiogenic or neurological problems, as they characterize the most likely differential diagnoses.

Furthermore, because the multilingual BERT model is used as the text feature extractor, the proposed model can search for free text in English or Chinese. The retrieval results included relevant symptoms and possible diseases, likely from the co-occurrence relationship [27] of structural data or the feature extracted from the multilingual BERT model. The multilingual BERT model was pretrained again by the chief complaint and present illness pair. Even if we only use the chief complaint with structured data and medical history as input, the characteristics of the chief complaint may reflect those patients with the same complaint but different diseases discovered by the doctor.

The potential of the semantic vector on the EHR search engine has already been demonstrated [27]. Our main contribution to the development of EHR retrieval is the construction of patient-level conceptual embeddings. However, in some cases, such as complaints about epileptiform (query 12) and shingles-like skin lesions (query 6) (see Multimedia Appendix 3), retrieval performance was poor. They would also result from a co-occurrence relationship with structural data or medical records. In future work, more EHR data could be used to extend the model, which may enrich the performance for differential diagnosis and retrieval.

This pretrained model is also suitable for finetuning the outcome prediction. For the National Hospital and Ambulatory Medical Care Survey, our pretrained model outperformed the reference. The pretrained method of disease concept-embedding can improve the performance of subsequent tasks. The outcome prediction performance we obtained for National Hospital and Ambulatory Medical Care Survey data was slightly higher than for data from the National Taiwan University Hospital Integrated Medical Database. This result may be related to the structured chief complaints and comorbidities in the National Hospital and Ambulatory Medical Care Survey data. These structural features had been well extracted by humans using rule-based methods. In future work, we will use all medical histories instead of their average values or preprocess them using extractive summarization methods.

We proposed a model that uses limited information for embedding. The model is a useful contribution, given that the input data can be collected by people without a medical background. For instance, a sick person who has no idea what occurred to him or her can still collect information and feed it into the model to obtain more information about his or her problem. Simultaneously, the model can also help hospital staff determine a patient’s disease with limited information. Most doctors (not only emergency department doctors) develop treatment plans based on limited information. Initially, doctors can obtain basic demographic information only after the patient has made an appointment. The doctor will then collect more information when the patient visits, including the chief complaint, symptoms, and medical histories.

Furthermore, physical examination and some blood laboratory examinations, radiology, or ultrasound examinations are arranged to help establish a diagnosis and finalize the treatment plan. The disease concept was initially established by limited information and prompted doctors to arrange specific examinations; the disease concept will become more prominent. Similarly, disease embedding obtained through limited information can only be used for preliminary differential diagnosis. It can be further applied to suggest the most relevant information for the final diagnosis. After more information is appended to the embedding model, a precise diagnosis could be identified.

Some EHR models use ICD codes as inputs. However, problems may arise; for example, the disease might not have the correct ICD code in EHRs [9], or ICD coding rules might change, invalidating the model trained on the old ICD codes. In this study, we did not use ICD codes as inputs. In addition to the time-varying ICD coding rules, ICD coding requires experts, which is difficult for people with no medical background. Sometimes, if there is not enough information, even experienced doctors have difficulty with ICD coding. However, doctors can collect more information during treatment and observation, and then determine the disease and choose the most appropriate ICD code. Consequently, some of the ICD codes recorded in an EHR may be future information and may not be suitable for input to predictive models in some retrospective studies.

For machine learning methods used to embed disease concepts, vast amounts of patient data are required. Therefore, protecting personal privacy is a crucial issue in the research process. A leak of a patient’s disease information would be disastrous and would infringe upon the patient’s legal rights. Although several studies have focused on this issue, no accurate quantitative method exists to assess the privacy of research data sets [2]. For private EHR data sets that have not been evaluated for de-identification quality, encoding the patient’s records and decoding them outside the hospital could create legal issues. In this study, DIM and SimCLR were used as the unsupervised methods, which have greater embedding capabilities [20] and do not train the decoder together, so there is no risk of leaking private information.

For the DIM method that maximizes mutual information, we assume each patient’s visit is unique. This assumption is also critical for the SimCLR method, because the self-supervised learning methods require negative samples to maximize the embedding distance among groups. If the same input exists in the negative sample, it will disrupt the training process and cause failure to converge. This assumption is reasonable because everyone is undoubtedly unique. The only problem arises when the same patient revisits a health care facility because of the same disease. However, this problem can be solved by deleting duplicate accounts in the data set. For patients with several accounts, these visits can still be regarded as different due to different health conditions, chief complaints, or previous medical histories. We also assume that the number of diseases is limited and fewer than all visits. This assumption is also reasonable because the total number of visits is set to the size of the training set, and in medical experience, at least two patients suffer from the same disease.

The SimCLR method proposed a simple method to use a large amount of negative samples for self-supervised learning. In this study, ideally, we would have randomly masked the tokens in the input free text data. However, owing to graphic processing unit memory limitations and the heavy BERT model, if an ideal augmented method is adopted for a positive sample, only 8 samples could be used in each mini-batch, thereby sacrificing the advantage of a large number of negative samples. Consequently, we ended up adding a little noise to each *m_i_* as a positive sample; in other words, we sacrificed the advantage of a positive sample.

Based on the ablation study results, both DIM and SimCLR can improve prediction performance through pretrained embedding. Although the improvement gap was small, the embedded pretraining method may be useful in future work. Furthermore, combining these two self-supervised methods can further improve prediction performance. These results are similar to those of DIM, which used two different discriminators on the training process and achieved a higher score [22].

### Limitations

Although the National Taiwan University Hospital Integrated Medical Database included one medical center and two regional hospitals and had many patients and staff with extensive disease treatment experience, there is still a problem of “out-of-disease,” which indicates that the disease is not in the EHR data set. This problem will result in poor performance for newly diagnosed diseases (eg, COVID-19), but diseases with related symptoms can be found for reference. Because the National Taiwan University Hospital Integrated Medical Database data set was not public, we only recruited one doctor to evaluate the relevance of the search results. Although the evaluation results were subjective, no objective score exists (according to previous studies) for evaluating information retrieval performance. We recognize that other evaluation methods in this study might have been more meaningful.

### Conclusions

The EDisease model uses limited information from patients and appropriately represents concept embedding. It can be further expanded as more data about the patient is collected.

The suitably-pretrained EHR model can be used as a medical experience retrieval system online in conjunction with the clinic staff. Moreover, it can be further finetuned to predict emergency events and enhance employees’ capabilities. The EDisease model could be widely adopted in the near future to help ease emergency department overcrowding: “ED-is-ease.”

## Appendix

Multimedia Appendix 1 Extended model.

Multimedia Appendix 2 Visualization of Embedding of Disease by t-SNE(2D). The embeddings were displayed in a list of (A) non-enhanced, (B) enhanced by gender, (C) age (10-year interval), (D) hospital level, (E) triage level, (F) whether a medical history exists, (G) outcome of ICU admission, and (H) death. There seems to be no specific difference in disease embedding based on (B) gender, (C) age> 10 years, (D) hospital grade, and (F) medical history. Triage level five, purple, was clustered together (E). In this disease embedding, the outcome of ICU or death did not appear to be linearly separable.

Multimedia Appendix 3 Disease Retrieval Relevance. The relevance of each result to the query was judged by a seasoned doctor, who used the number of hits on the top five results to score from zero to five. Retrieval performance was high, but in some cases, such as shingles-like skin lesions (query 6) and epileptiform (query 12), the retrieval performance was poor.

Multimedia Appendix 4 Disease Retrieval Demonstration. The query subject (orange) was a 63-year-old male patient who suffered from sudden nausea, vomiting, and weakness and felt facial numbness at work. Through the retrieval, we obtained the top five similar patients (green) whose symptoms were palpitation, chest pain, nausea, dizziness, anorexia, insomnia, and dizziness combined with limbs tremor. GCS: Glasgow coma scale. SBP: Systolic blood pressure. DBP: Diastolic blood pressure. HR: Heart rate. SpO_2_: Blood oxygen saturation. RR: Respiratory rate. BT: Body temperature in Celsius. BH: Body height. BW: Body weight. PS: Pain scale. E: Eye response in Glasgow coma scale. V: Verbal response in Glasgow coma scale. M: Motor response in Glasgow coma scale. N/A : Not available.

## Abbreviations

AUROCs: Area Under the Receiver Operating Characteristics
BERT: Bidirectional Encoder Representations from Transformers
DIM: Deep InfoMax
EHRs: electronic health records
ICD-10: International Statistical Classification of Diseases and Related Health Problems 10th Revision
SimCLR: Simple Contrastive Learning of Visual Representations
t-SNE: t-distributed stochastic neighbor embedding
