# Supplementary figures and images for: Disease Concept-Embedding Based on the Self-Supervised Method for Medical Information Extraction from Electronic Health Records and Disease Retrieval: Algorithm Development and Validation Study
Source: J Med Internet Res. 2021 Jan 27;23(1):e25113. doi: 10.2196/25113 (PMC7875703; doi:10.2196/25113)

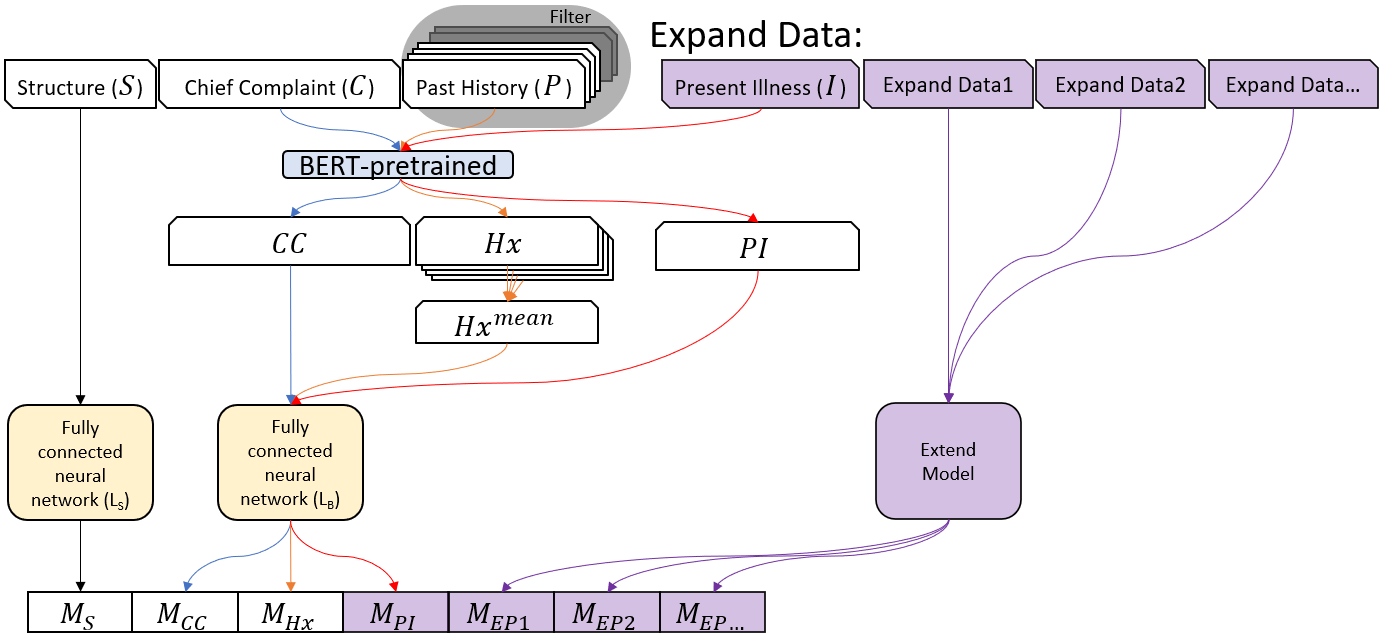

Supplement: Multimedia Appendix 1 [file jmir_v23i1e25113_app1.png]

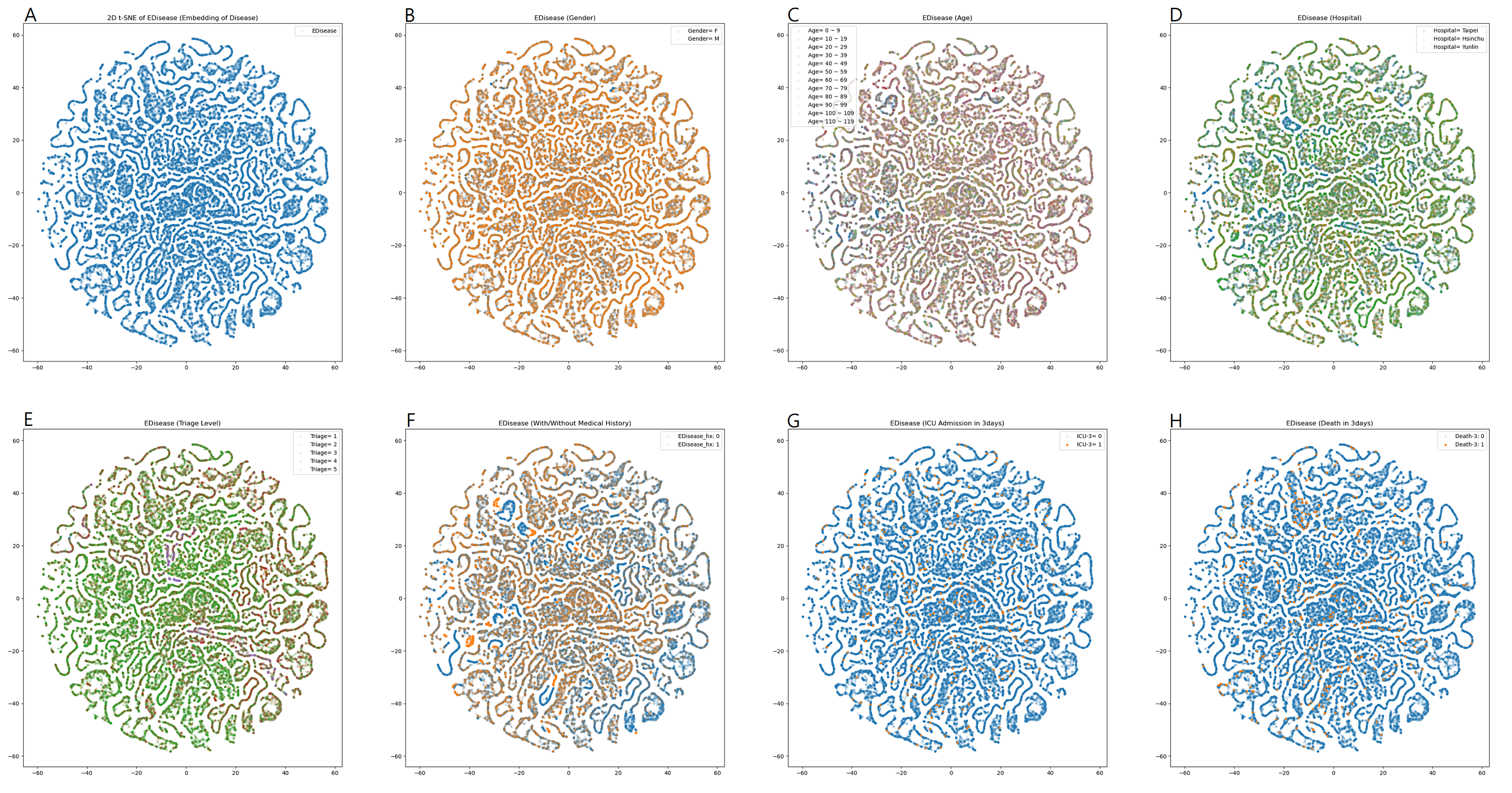

Supplement: Multimedia Appendix 2 [file jmir_v23i1e25113_app2.png]

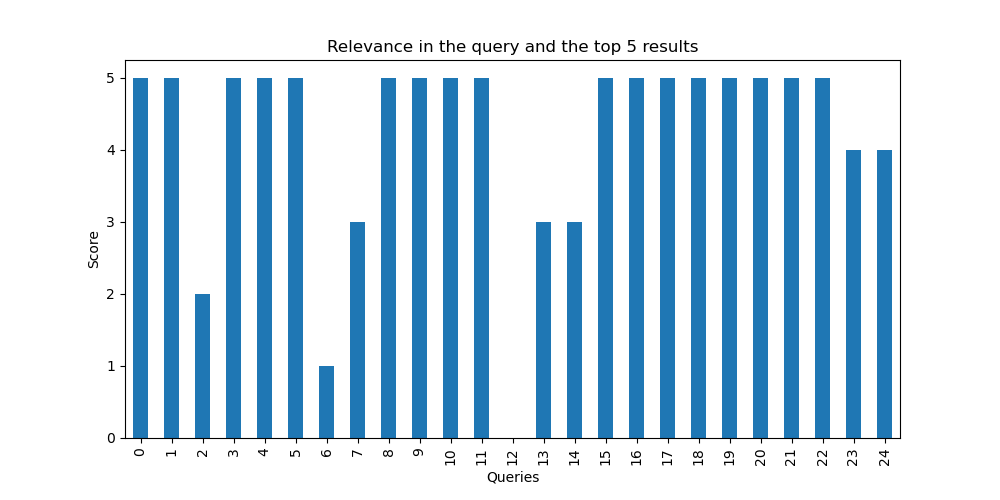

Supplement: Multimedia Appendix 3 [file jmir_v23i1e25113_app3.png]

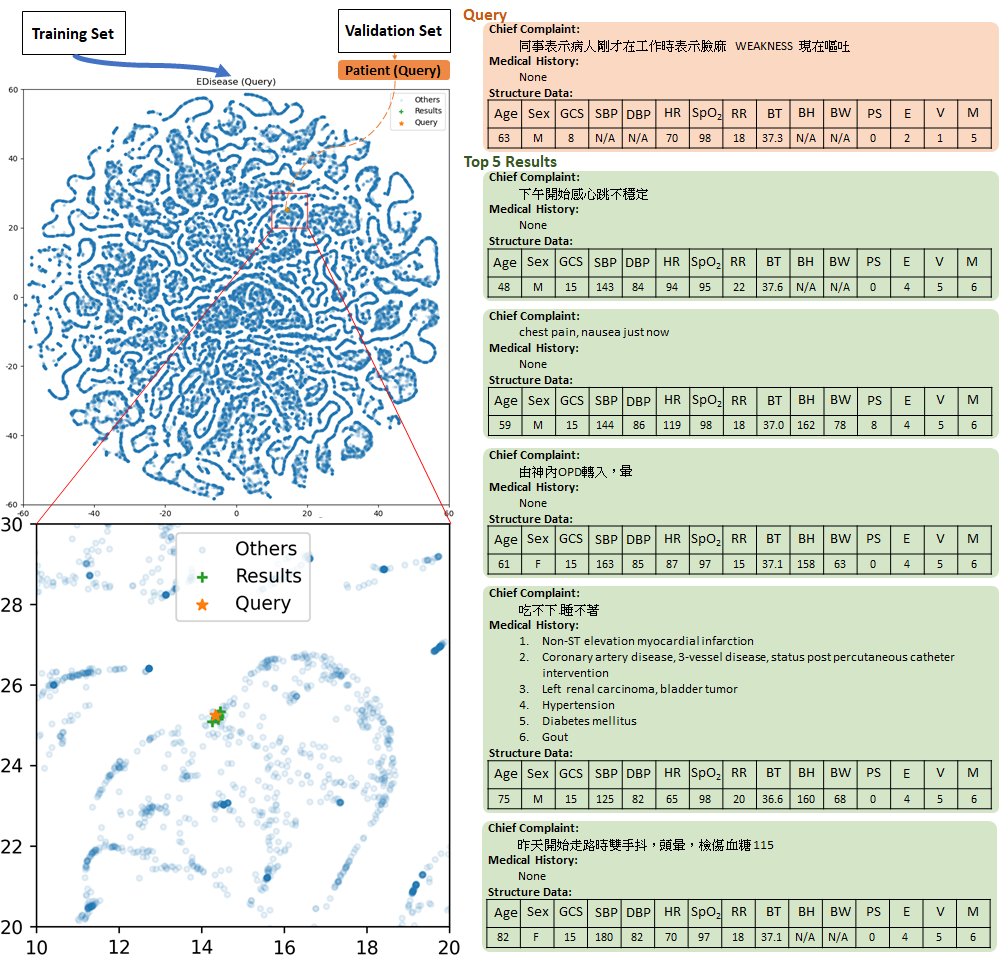

Supplement: Multimedia Appendix 4 [file jmir_v23i1e25113_app4.png]
